# Numerical Model to Simulate Electrochemical Charging of Nanocrystal Films

**DOI:** 10.1021/acs.jpcc.3c01562

**Published:** 2023-05-15

**Authors:** Reinout F. Ubbink, Solrun Gudjonsdottir, Yan B. Vogel, Arjan J. Houtepen

**Affiliations:** Optoelectronic Materials Section, Faculty of Applied Sciences, Delft University of Technology, Van der Maasweg 9, Delft 2629 HZ, The Netherlands

## Abstract

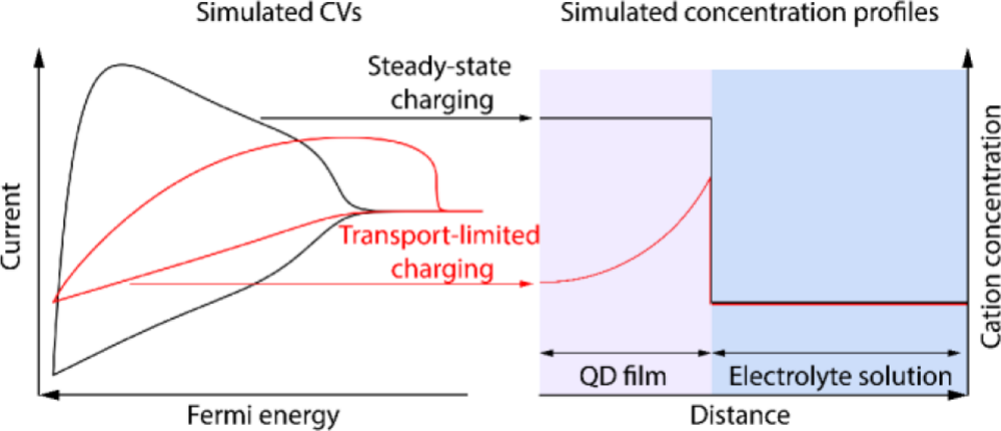

Electrochemical charging of nanocrystal films opens up new possibilities for designing quantum dot-based device structures, but a solid theoretical framework of this process and its limitations is lacking. In this work, drift-diffusion simulations are employed to model the charging of nanocrystal films and gain insight into the electrochemical doping process. Through steady state simulations it is shown that the Fermi level and doping density in the nanocrystal film depend on the concentration of the electrolyte in addition to the value of the applied potential. Time-resolved simulations reveal that charging is often limited by transport of electrolyte ions. However, ion transport in the film is dominated by drift, rather than diffusion, and the concentration profile of ions differs substantially from concentration profiles of diffusing redox species at flat electrodes. Classical electrochemical theory cannot be used to model this type of mass transport limited behavior in films of nanocrystals, so a new model is developed. We show that the Randles–Ševčík equation, which is derived for electrochemical species diffusing in solution, but is often applied to films as well, results in a significant underestimation of the diffusion coefficients of the charge compensating electrolyte ions.

## Introduction

Semiconductor nanocrystals, also called quantum dots (QDs), show promise in various optoelectronic applications, such as displays, LEDs, photodetectors, and lasers.^1−4^ The ability to control doping density in QDs is an important tool for the design of these applications as it can be used to increase the conductivity of QD films, lower the gain threshold of QD-based lasers, or create p-n junctions inside the films.^5^ Compared to traditional bulk semiconductors, impurity doping of QDs is challenging due to the formation of lattice defects and charged surface states, making precise control over the doping density hard to achieve.^6−8^ Electrochemical doping of QD films has been shown to be a viable alternative to impurity doping^9−11^ and can be used to simply and precisely control the Fermi level in a QD film. Additionally, no lattice distortion takes place as dopants are drawn from the electrolyte solution and are present externally in the voids between QDs in the nanoporous film.

While electrochemical doping of QD films has been successfully performed, a solid understanding of the process is still lacking. Various assumptions that hold true for electrochemistry on flat electrodes are often made for experiments on nanoporous electrodes such as QD films and battery electrode materials,^12−16^ but their validity for these systems is not obvious. In this work, we present a drift-diffusion simulator that is able to simulate a broad range of electrochemical measurements on nanoporous electrode materials. Using this simulator, we test the validity of some commonly made assumptions regarding the charging of these materials.

The first assumption that is often made is that the charge carrier concentration inside the nanoporous films is constant and that the Fermi-level is equal to the applied electrochemical potential. Through steady state simulations, we show the presence of two sharp potential drops at the interfaces of electrochemically doped semiconductor films. The first is due to an electrical double layer at the working electrode (WE)/QD film interface and is responsible for the change in Fermi level in the QD film. The second however forms at the QD film/solution interface. The presence of this potential drop implies that not all of the applied potential changes the Fermi-level inside the QD film. At an electrolyte concentration of 0.01 M, the film-solution potential drop can be as large as 10% of the total potential. At high electrolyte concentrations (>1 M), this potential drop can be minimized to <1% of the applied potential.

We then compare simulated cyclic voltammograms (CVs) to experimentally obtained CVs of ZnO QD films. At high scan rates, a square root relation between the scan rate and the current density is observed, indicating that the current is limited by mass transport of the cations inside the QD film. Analysis of the concentration gradient reveals that, unlike in flat electrode systems, the Randles–Ševčík equation does not hold when considering charging of a nanoporous electrode. Specifically, when an electrode material that is permeable to ions, using the Randles–Ševčík equation to calculate the diffusion coefficient of these ions leads to an underestimation of 1–2 orders of magnitude.

## Methods

**Figure 1:**
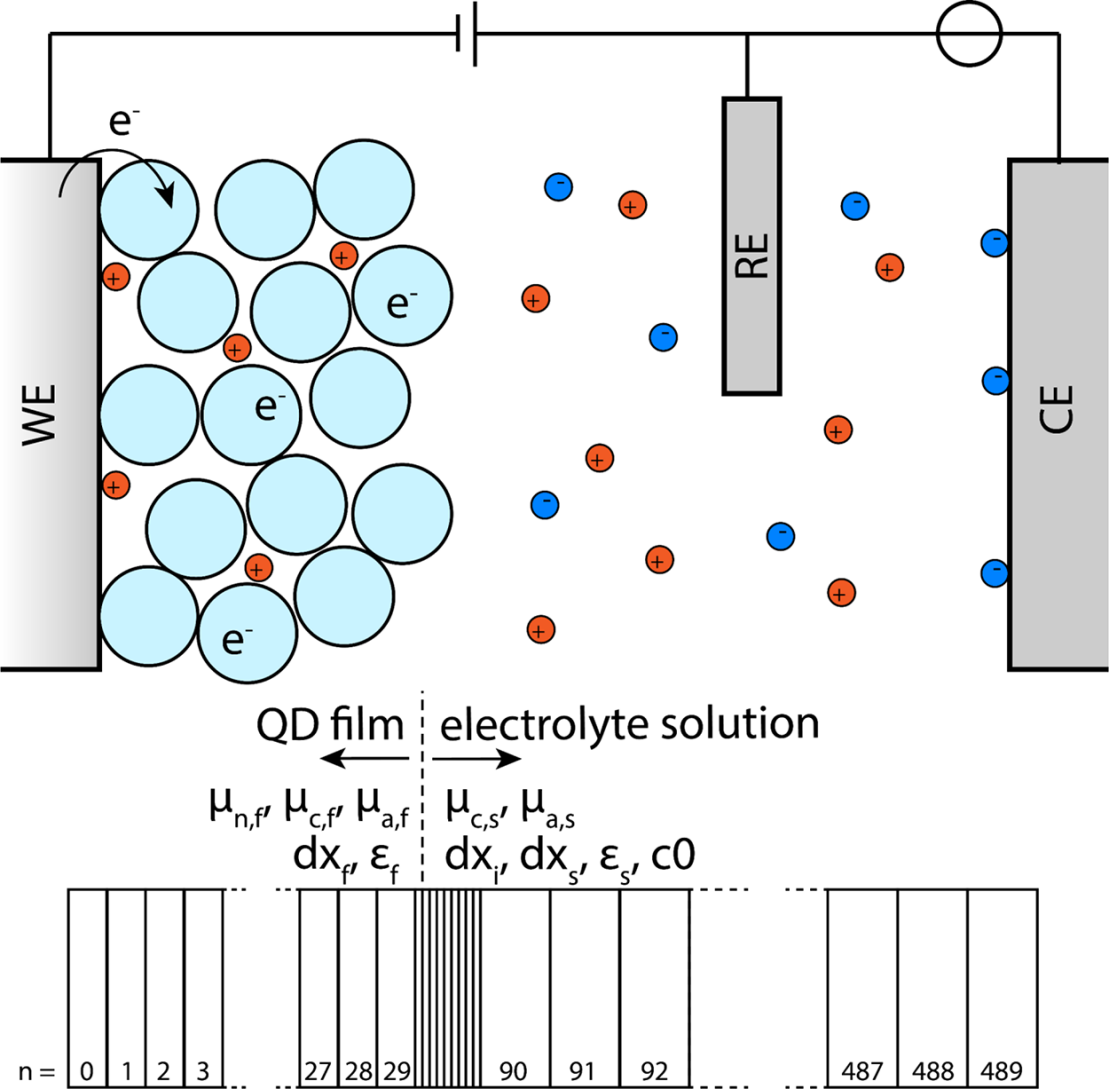
Schematic of the experimental three-electrode setup and the one-dimensional model used in the simulations. The WE and CE mark the edge of the simulated space, while the RE is placed in the middle of the electrolyte solution between the film/solution interface and the CE.

The simulator was inspired by work on light-emitting electrochemical cells by van Reenen et al.,^17^ but was redesigned to mirror a three-electrode electrochemical cell. An electrochemical cell with QD films on the WE is treated as a one-dimensional system, divided numerically in multiple (∼500) lamella, as shown in Figure 1. The one-dimensional simulated space starts at the WE, encompasses the QD film and the electrolyte solution, and ends at the counter electrode (CE). The reference electrode (RE) is positioned halfway between the film/solution interface and the CE. To achieve high spatial resolution in the film/solution interface region without sacrificing computational performance, the space was divided in lamella nonlinearly. The simulator considers an initial state, which contains starting values of the concentrations of electrons, anions, and cations for each lamella. It then determines the movement of these three charge carriers over small time steps based on the drift-diffusion equations (see Table S1, Supporting Information). The hole concentration is assumed to be zero as only negative applied potentials relative to the open circuit potential are considered in this work. During each time step, a midpoint method is used to solve the Poisson equation (Table S1) and to determine the spatial profile of the electrostatic potential for the next step. Boundary conditions of a regular three-electrode system are enforced, i.e., ϕ_WE_ – ϕ_RE_ = *V*_applied_ and ϕ_RE_ = 0 (with ϕ the electrostatic potential at a certain position), while the electrostatic potential at the CE is allowed to float. The true potential at the RE (and thus the initial Fermi level) at open circuit potential is arbitrary for the simulation, but for comparison with experiments was set to −4.7 V *versus* vacuum = 0.26 V *versus* SHE = −0.3 V *versus* ferrocene/ferrocenium (Fc/Fc^+^) in acetonitrile. This value corresponds to the work function of indium tin oxide (ITO) (used as WE) and is in accordance with the open circuit potential of experimental ZnO QD films (Figure S1 in the Supporting Information).^9,10^ The proficiency of the program in simulating the electrochemical behavior of three-electrode systems was confirmed by simulating a CV of a simple reductant/oxidant pair in Nernst equilibrium (Figure S2 in the Supporting Information). In the actual QD film simulations, the initial state of the simulation always consisted of an uncharged QD film, where the concentration of electrons was zero, and an electrolyte solution with a certain concentration of cations and anions, *c*_0_. An infinite supply of ions was achieved by setting the concentration of both ions at the RE constant at *c*_0_ during the simulation. The QD film was considered to have a porosity of 50%, with the pores filled with electrolyte solution at concentration *c*_0_. The electron concentration in the first lamella of the film, in contact with the ITO electrode, is governed by a Fermi–Dirac equilibrium with the electrode:
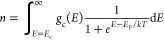
1where *n* is the concentration of electrons in the first lamella, *g*_c_(*E*) is the density of states (DOS) function of the material, *E*_c_ is the conduction band energy level, and *E*_F_ is the Fermi level in the first lamella, which is equal to the intrinsic Fermi level minus the electrostatic potential (ϕ) in the first lamella. Thus, as the applied potential becomes more negative, the value of the Fermi level, Fermi–Dirac integral, and the concentration of electrons all increase in the first lamella. Any DOS function can be used as input in the simulator. The simulation parameters, including the DOS function, were set at the start of the simulation after which only the applied potential was altered to obtain the CV curves shown in this work. Parameters used were optimized to most closely fit experimental data of electrochemical charging of ZnO films (see Table S2, Supporting Information). Parameters used here were kept consistent for every figure shown unless otherwise noted, but any set of input parameters can be used. For performance reasons, the simulator was written in C++ and compiled using Microsoft Visual Studio. Simulations were typically completed in a few minutes (for a scan rate of 1 V/s) to an hour (for a scan rate of 0.1 V/s and steady-state simulations) on a single core of a personal computer. Additional computational details, equations, DOS functions, and a list of parameters that were used in these simulations can be found in the Supporting Information. The simulator source code and accompanying instructions are available at GitHub: github.com/RFUbbink/QDfilmsim.

## Results and Discussion

### Drift-Diffusion Simulator

For most QD materials, experimental n-doping of QD films has proven more successful than p-doping.^10,11,18−21^ For this reason, we have only considered negative applied potentials (relative to the open circuit potential), electron injection and n-type doping, while neglecting the presence of holes, although extension to hole injection would be straightforward. Since the movement of both electrons and ions is predicted by drift-diffusion theory, this is an ideal basis for simulating the electrical behavior of QD films in an electrochemical cell.

We designed the one-dimensional simulator to mirror a three-electrode electrochemical cell, as shown in Figure 1. The simulator consists of three sections: the QD film, the film/solution interface, and the electrolyte solution. The QD film is in contact with the WE, while the electrolyte solution is in contact with both the RE and the CE. The film/solution interface plays a critical role in determining the electrical response and is therefore deserving of its own separate section. Cations and anions can freely flow anywhere in the simulation according to drift-diffusion theory, but electrons are confined to the QD film. When negative potentials relative to the open circuit potential are applied to the WE, cations migrate toward it and an electrical double layer (EDL) develops at the WE/QD film interface. In the EDL (typically ∼1.5 nm wide), the electrostatic potential (ϕ) drops as the excess cations shield the negative charge of the WE. This potential drop at the WE/film interface (Δϕ_E/F_) increases as the applied potential becomes more negative. The presence of Δϕ_E/F_ leads to band bending in the QD film (see Figure S3 in the Supporting Information) and a decrease of the QD conduction band edge relative to the work function of the electrode. In other words, the Fermi level of electrons in the QD film is raised (Figure S3). If the applied potential is negative enough to raise the Fermi level above the conduction band edge, electrons will be injected from the WE into the conduction band of the QDs, while additional cations will flow from the solution into the voids of the QD film to compensate the excess negative charge.

### Steady State Concentration and Potential Profiles

**Figure 2:**
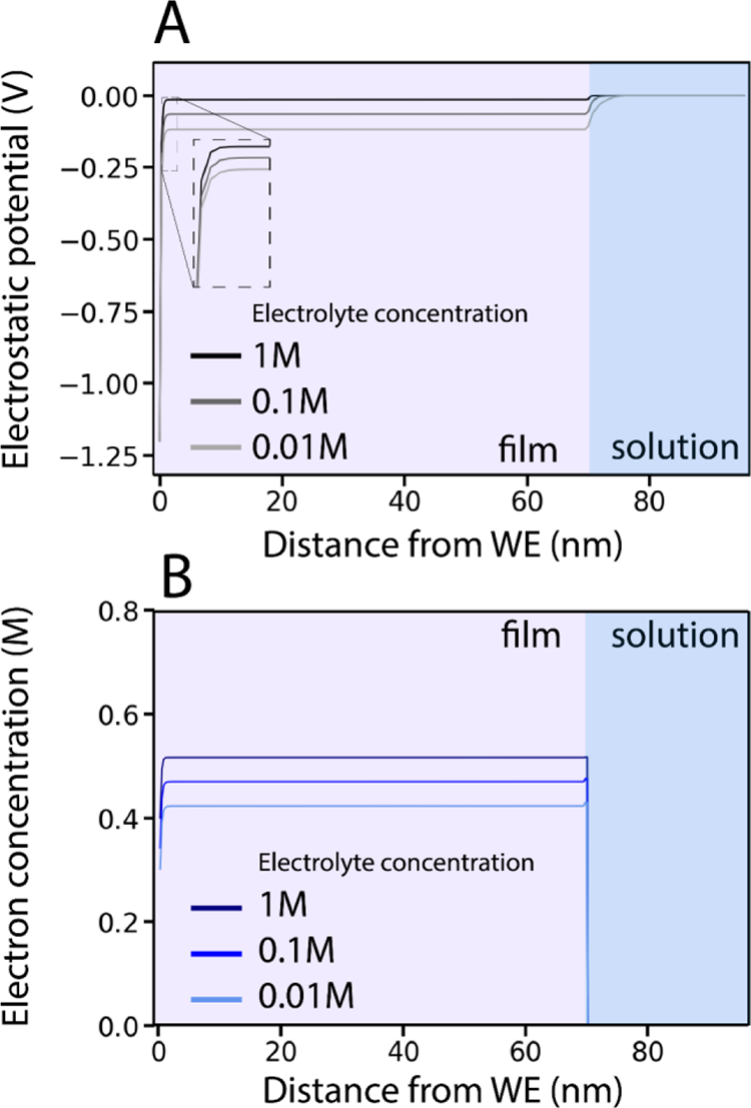
(A) Simulated steady state potential and (B) simulated electron concentration profiles for electrolytes with different ion concentrations for an applied potential of −1.2 V. The film–solution interface is indicated at 70 nm. Both the potential and electron concentration are constant over the remainder of the electrolyte solution, which has been omitted for clarity. Electron concentration is constant throughout the bulk of the film but deviates slightly at the edges due to the EDLs.

Steady state solutions of the three-electrode system were obtained by running the simulator at an applied potential of −1.2 V *versus* the RE until the current was <0.1% of the maximum current. The electrostatic potential profile and electron concentration profile obtained for three different electrolyte concentrations in this way are depicted in Figure 2.

At steady state, the electron concentration in the film is constant and equal to the excess cation concentration in the film: electrochemical n-doping has been achieved at a constant concentration throughout the film. The electrostatic potential over the film is also constant, but two sharp drops of the electrostatic potential are observed at the interfaces of the film (Figure 2A). The aforementioned potential drop at the WE–film interface develops as cations are attracted to the negative charge in the electrode and accumulate in an electric double layer. This potential drop is the reason electrons can be injected, as it provides the potential energy necessary to overcome the injection barrier between the electrode work function and the conduction band of the QDs. As Δϕ_E/F_ grows larger, electrons can be injected into higher energy states, thus increasing the equilibrium concentration of electrons in the film (Figure S3).

The second potential drop, at the film–electrolyte solution interface (Δϕ_F/S_), is responsible for providing the excess concentration of cations in the film that is necessary to compensate the charge of the electrons. Without Δϕ_F/S_, ions would diffuse back into the solution and no steady state would be achieved. Migration due to Δϕ_F/S_ balances this diffusion at steady state. High spatial resolution (d*x_i_* < 0.5 nm) is needed to simulate Δϕ_F/S_ accurately. Low resolution leads to an overestimation of the potential drop: as the entire drop will be contained in one lamella, increasing d*x_i_* will result in the same electric field being considered over a longer distance during integration and thus an unphysically high interface drop. A comparison of interface potential drops calculated from simulations at different resolutions can be found in Figure S4. Increasing the resolution from 0.3 to 0.2 nm leads to only a 3% change in Δϕ_F/S_.

To achieve high spatial resolution at the interface without increasing the number of lamella, a variable lamella thickness was employed in the simulators (each of the three sections has its own d*x* value). For the steady state simulations in Figure 2, an interface resolution of 0.1 nm was used, while for the transient simulations below a resolution of 0.3 nm was used, achieving good accuracy without compromising the performance of the simulator.

As can be seen in Figure 2A, for a 1 M concentration of ions in the electrolyte solution, Δϕ_F/S_ is ∼1% of the total applied potential and practically the entire potential drops at the WE–film interface. This means that the Fermi level in the semiconductor film is raised from the intrinsic level by an amount roughly equal to the applied potential.

At lower electrolyte concentrations, however, a larger potential drop is observed. At 0.01 M about 10% of the potential drops at the electrolyte solution interface. In these low concentration conditions, the increase in Fermi level in the film is no longer equal to the applied potential. If Δϕ_F/S_ is larger, Δϕ_E/F_ is smaller for the same applied potential, thus resulting in a lower electron concentrations and lower doping density (Figure 2B). Doping densities in these simulations range from 0.51 to 0.42 M for electrolyte concentrations of 1 and 0.01 M, respectively (Figure S5), a difference of 21%.

To achieve maximum doping density in QD films, it is therefore favorable to increase the concentration of the electrolyte solution. This is also important when thermodynamic properties are derived from electrochemical measurements, such as the density of electron states. This effect of the QD-film/solution interface has previously been overlooked.

As long as steady state systems are considered, it is possible to calculate the minimum electrolyte concentration needed to charge a nanoporous film to a certain doping density while keeping Δϕ_F/S_ below a chosen threshold (see also derivation S1 in the Supporting Information). When steady state has been reached, the concentration of cations in the system follows the Boltzmann distribution:
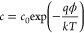
2where *c* is the cation concentration, *c*_0_ is the bulk electrolyte concentration (assuming an infinite supply of bulk cations), *q* is the elementary charge, ϕ is the electrostatic potential, *k* is the Boltzmann constant, and *T* is the temperature. This relation is observed in the steady state simulations shown in Figure 2. Rewriting this distribution yields:
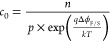
3where *n* is the electron concentration in the nanoporous film and *p* is the porosity of that film. For example, for a film (porosity = 50%) of QDs with a diameter of 3.5 nm (QD volume = 22 nm^3^, QD “concentration” = 0.0742 M), if one wants to charge the film with 8 electrons per QD while keeping Δϕ_F/S_ < 2 *kT* (=0.052 eV at RT), this would require a minimum electrolyte concentration of 0.16 M:
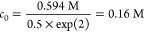
4

### Cyclic Voltammetry Simulations of QD Films

**Figure 3:**
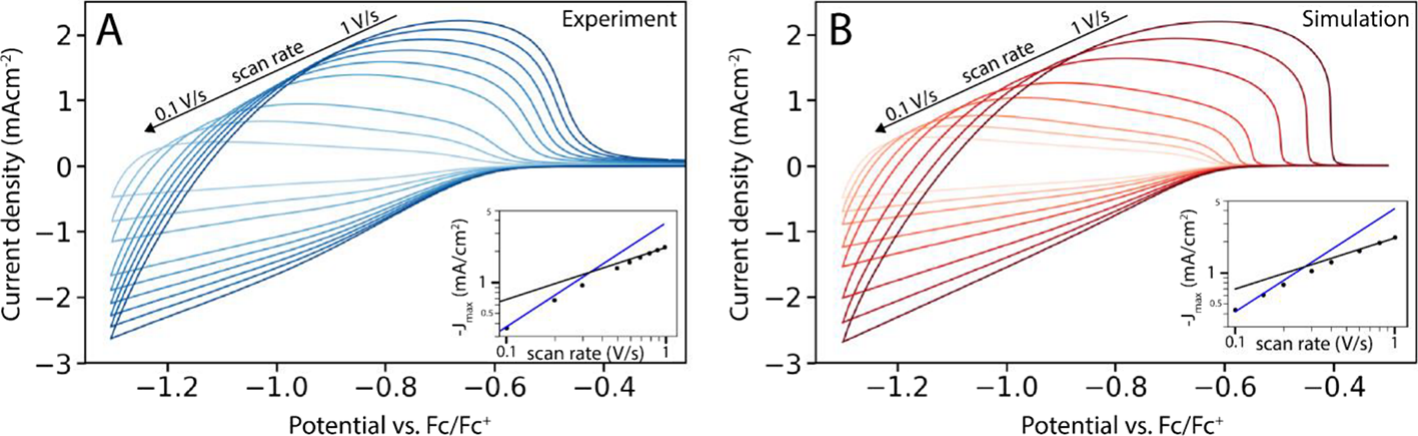
Comparison of experimental (A) and simulated (B) CVs of QD films. Experimental data were collected in a three-electrode cell, employing a ZnO QD film (thickness = 700 nm) on top of an ITO WE, and a 0.1 M LiClO_4_ acetonitrile electrolyte solution. For more experimental details see ref (9). Excellent agreement between simulation and experiment is observed for a range of scan rates. Insets show the relationship between the maximum current and the scan rate. In the insets, a transition from capacitance-limited charging (slope = 1, blue line) to mass transport-limited charging (slope = 0.5, black line) is observed as the scan rate is increased in both the experimental and simulated CVs.

To acquire information on the dynamics of charge injection into QD films, cyclic voltammetry is commonly employed. Figure 3A shows such experimental CVs on a film of ZnO QDs at different scan rates, collected and shown in prior work by Gudjonsdottir et al.^9^ To analyze these CVs, the scans were also simulated. The applied potential was changed over time and the current was recorded by counting the amount of electrons that entered the QD film from the WE. Parameters were chosen to mirror those of the experiments on ZnO QD films, and then optimized to fit to the experimental data (Table S2). A set of simulated CVs at different scan rates is shown in Figure 3B. With a single parameter set, excellent agreement with experimental data is achieved across a wide range of scan rates.

In a typical CV of a QD film, the current density is small (<1 μA/cm^2^) and attributed to the formation of the EDLs at the electrodes until the applied potential is high enough to inject electrons into the conduction band of the QDs (around −0.6 V vs Fc/Fc^+^ in these simulations). Before this point, the Fermi level is still in the band gap of the QDs. Once the Fermi level is raised above the conduction band edge, significant n-type doping starts to occur, and a negative current starts to flow. After the applied potential has reached its minimum and the scan direction is inverted, a positive current is observed as electrons are extracted from the QDs and flow back into the WE.

The electrical response of a QD film during a CV is strongly dependent on the scan rate. When a low enough scan rate is used (for example, 0.1 V/s for a film thickness of 700 nm), the current density is determined by the capacitance of the film. In this case, a linear relationship between the peak current density and scan rate is expected, since the same amount of charge is injected regardless of the scan rate.^9^ In both the experiments and simulations, this linear relation is observed for low scan rates (insets Figure 3A,B). Under these conditions, the simulations show that the concentration profiles of electrons and cations over the film are nearly constant, indicating that the film is charged with electrons in near-steady state conditions. When low scan rates are applied, the shape of the CV is expected to almost perfectly trace the DOS of the QDs (save for the potential drop over the film solution interface discussed above). This can be simulated by adjusting the DOS function.

**Figure 4:**
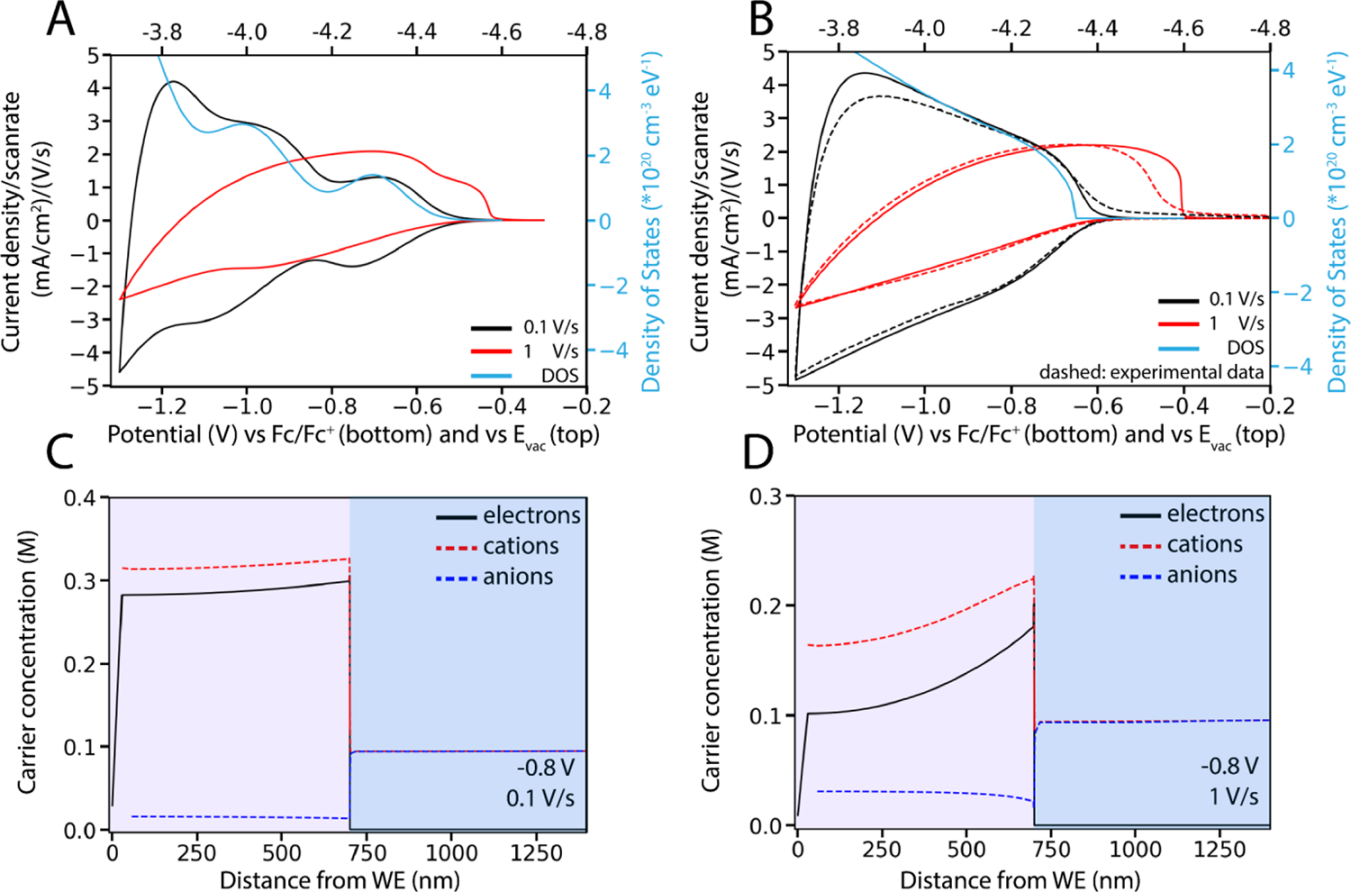
(A and B) Comparison of simulated CVs and the corresponding concentration profiles of QD films with different scan rates and the corresponding DOS functions (blue) that were used in the simulation, at an electrolyte concentration of 0.1 M. At a scan rate of 0.1 V/s, the CV shape is mostly determined by the shape of the DOS. The concentration profile that is observed in this near-steady state regime is visible in (C). At higher scan rates, the current density is limited by carrier mobility and the CV shape is mostly independent of the DOS function of the material. In this case, the current is limited by cation transport, and an accompanying concentration profile is observed (D).

Figure 4A,B depicts simulated CVs of materials using a different DOS function. At a scan rate of 0.1 V/s, the two CVs differ significantly, tracing the shape of the corresponding DOS function. The DOS used in Figure 4A was chosen to resemble a typical QD DOS function, featuring 3 Gaussian peaks for the s, p, and higher energy levels. The corresponding simulated CV resembles experimental CVs observed for CdSe QDs.^11^ However, experiments on ZnO QD films result in CVs with a distinctly different shape.^9,10^ To best fit the experimentally observed results, we chose a DOS function that resembled that of a bulk semiconductor (square root function of energy) combined with some minor QD features (Gaussian peaks). Using this DOS we were able to closely reproduce experimental CV shapes (Figure 4B). This is in accordance with earlier results from Brozek and colleagues, which indicated that discrete energy levels were not visible in the ensemble capacitance of ZnO QD films,^22^ and generally shows that in these drop-cast and mildly annealed ZnO QD films quantum confinement is weak.

As long as low enough scan rates are applied, the shape of simulated and experimental CVs shows a strong correlation with the DOS function of the material that is used, and simulations can be used to extract information on the DOS function of QD materials. Qualitative DOS functions of QD materials can be extracted by fitting experimental CVs as shown in Figure 4, and even quantitative DOS values can be obtained from CVs at low scan rates if the number of QDs in the film or the film thickness and porosity are known. In Figure 4B, the quantitative DOS function that was fitted for a ZnO QD film is plotted in cm^–3^ eV^–1^.

At higher scan rates, however (1 V/s for a film thickness of 700 nm is used in Figure 4), the shape of the CV is mostly independent of the DOS function of the material. The current density is limited instead by the mobility of one of the charge carriers. Charge carrier mobility is related to the diffusion coefficient through the Einstein relation:
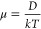
5where μ is the mobility of a charge carrier and *D* is its diffusion coefficient. Earlier work showed that electrons are typically very mobile in ZnO QD films (electron mobility of ∼0.1 cm^2^/V s), while cation movement through the pores of the film is slow (mobility of Li^+^ is ∼10^–7^ cm^2^/V s) and limits the speed of charge injection.^9,23^

In Figure 4C, it is shown that at low scan rates, the concentration profiles of cations, anions, and electrons in the film resemble those obtained in the steady state simulations: the concentration of both electrons and cations is almost constant throughout the QD film. At low scan rates (0.1 V/s), the film is charged in near-steady state conditions.

When faster scan rates are applied (1 V/s), cation transport is too slow to achieve constant concentrations, resulting in the concentration profile shown in Figure 4D. This concentration profile that develops is quite different from the concentration profile that develops in electrochemical experiments on redox active molecules in solution using flat electrodes. For such flat electrode systems, it is well-known that the peak current in the CV, *i*_p_, can be related to the mobility of the electrochemical species though the Randles–Ševčík equation:^24−26^
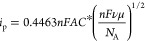
6where *n* is the number of electrons involved in the reaction, *F* is the Faraday constant, *A* is the area of the WE, C* is the concentration of the redox active molecule, ν is the scan rate, and μ is the mobility of the redox active molecule. If the Randles–Ševčík equation holds, a square root-relationship between the scan rate and peak current density is expected. Such a relationship is indeed observed at faster scan rates in both the experimental and simulated scan rate series presented in Figure 3 (black line in the insets). For this reason, we have earlier used the Randles–Ševčík equation to estimate the mobility of cations in the QD film.^9,19^ The Randles–Ševčík equation is also commonly used to determine diffusion coefficients in, e.g., battery research, where a similar process is considered: the diffusion of ions into an electrode material where their charge is compensated by electrons.^12,14−16^ Using the drift-diffusion simulator, we can now assess the validity of this equation for the case of charge-compensated electron injection into nanoporous electrodes, such as, a battery electrode or a QD film.

In Figure S6, a series of mobilities as calculated by the Randles–Ševčík equation is compared with the actual mobilities as they were set at the start of the simulation. Using the equation on the CVs obtained in the simulation yields a cation mobility that is a factor 10–300 lower than the real mobility, showing that the Randles–Ševčík equation is not valid for the charging of a nanoporous film system.

This can be explained by considering the cation and electron concentration profiles more closely: since the movement of cations into the film is limiting, the cation concentration must be higher near the film/solution interface and lower near the WE. In order to maintain local charge neutrality, electron concentrations must necessarily follow the same profile. By solving the drift-diffusion equations for this system, it can be shown that the concentration profile of cations and electrons increases quadratically with distance from the WE/film interface (see Table S3 and derivation S2 in the Supporting Information), which is different from the concentration profile observed in flat-electrode electrochemistry.

Furthermore, since both cations and electrons follow the same concentration profile (Figure 4D), mass transport cannot be completely diffusion controlled: if only diffusion currents were present in the film, both cations and electrons would diffuse toward the WE, while the direction of total electron flux must be toward the film/solution interface during electrochemical charging. In the simulations, electric fields are indeed observed, which result in a drift current inside the film that is 11 times stronger than the diffusion current (Figure S8). While charging is still limited by the transport of the cations, drift currents dominate in the film, rather than diffusion.

An assumption necessary for the application of the Randles–Ševčík equation is that drift current is negligible and transport of redox species is fully diffusion-controlled. This is a reasonable assumption for flat-electrode systems as long as the concentration of the supporting electrolyte is significantly higher than that of the redox species. In that case, the supporting electrolyte will form a thin EDL at the electrode surface, and transport of the redox species takes place outside the EDL and is completely diffusion-controlled. Because of the electric fields present inside the film during electrochemical charging of nanoporous films, the system is significantly different and the Randles-Ševčík equation is not applicable.

We note that the analysis given here only holds when considering the electrochemical charging of an electrode material, i.e., charged ions and electrons or holes enter and remain in the electrode material itself. Purely heterogeneous electrochemical reactions on (nano)porous electrodes are subtly different, and the Randles–Ševčík equation is applicable in those cases when a supporting electrolyte is present as shown in earlier simulations by Henstridge et al.^27^

## Conclusions

One-dimensional drift-diffusion simulations were found to accurately reproduce electrochemical experiments on the charging of QD films, showing that drift-diffusion theory is an excellent model to describe the movement of charge carriers in these systems. Through steady state simulations, it was revealed that the electrostatic potential drop is distributed over the film/electrode interface and the film/solution interface, with a ratio that depends on the electrolyte concentration. This implies that the doping density that can be achieved in nanoporous films through electrochemical charging depends both on the applied potential and the concentration of electrolyte ions. It was further shown that the DOS function of QD materials can be obtained by combining simulations and experimental CVs at low scan rates. When QD films are charged at high scan rates, cation transport in the QD film limits the charging speed. Contrary to earlier discussions on this topic we show that this cation transport is dominated by drift, rather than diffusion, and that the concentration profile of ions is very different from that for electrochemical reactions in solution. We show that the often-used Randles–Ševčík equation does not hold for the charging of nanoporous electrodes but underestimates the actual ion mobility by 1–2 orders of magnitude.

## Supporting Information Available

Computational details, simulation parameters, additional simulation data, and mathematical derivations (PDF)
