# Supplementary material for: Numerical Model to Simulate Electrochemical Charging of Nanocrystal Films
Source: J Phys Chem C Nanomater Interfaces. 2023 May 15;127(20):9896–902. doi: 10.1021/acs.jpcc.3c01562 (PMC10226107; doi:10.1021/acs.jpcc.3c01562)
Supplement: Supplementary file 1 — jp3c01562_si_001.pdf [file jp3c01562_si_001.pdf]

Supporting information for

Numerical Model to Simulate Electrochemical Charging of Nanocrystal Films

*Reinout F. Ubbink†, Solrun Gudjonsdottir, Yan B. Vogel†, Arjan J. Houtepen†**

† Optoelectronic Materials Section, Faculty of Applied Sciences, Delft University of Technology, Van der Maasweg 9, 2629 HZ Delft, The Netherlands

Table S1. List of formulas employed in the simulator.

Formula	Name and purpose	Explanation of parameters
$J_n = nq\mu_n \frac{dV}{dx} - kT\mu_n \frac{dn}{dx}$<br>$J_c = cq\mu_c \frac{dV}{dx} + kT\mu_c \frac{dc}{dx}$<br>$J_a = aq\mu_a \frac{dV}{dx} - kT\mu_a \frac{da}{dx}$	**Drift-diffusion equations**<br>Calculate the currents of electrons (n), cations (c) and anions (a).	J: current density<br>q: elementary charge<br>V*: electrostatic potential level vs vacuum<br>μ: carrier mobility
$\frac{d^2V}{dx^2} = \frac{q}{\varepsilon_0 \varepsilon_r}(n + a - c)$	**1D Poisson equation**<br>Cal+culate the potential profile over the space of the simulation.	ε_0, ε_r: vacuum and relative electric permittivity.
$n = \int_{E=Ec}^{\infty} g_c(E) \frac{1}{1 + e^{\frac{E - E_F}{kT}}} dE$	**Fermi-Dirac distribution**<br>Calculate the equilibrium concentration of electrons at the interface between the WE and the QD film.	E: energy<br>E_c: conduction band level<br>E_F: current Fermi level (= intrinsic Fermi level - electrostatic potential)<br>g_c(E): density of states function of the material

* For electrons, the total potential relative to vacuum is instead used, i.e. *energy level in the DOS* vs. *vacuum* + *electrostatic potential*. In this way, the extra energy needed for electrons to occupy higher levels in the DOS at higher energies is taken into account in calculating the drift current. The position of electrons in the DOS is calculated from the electron concentration.

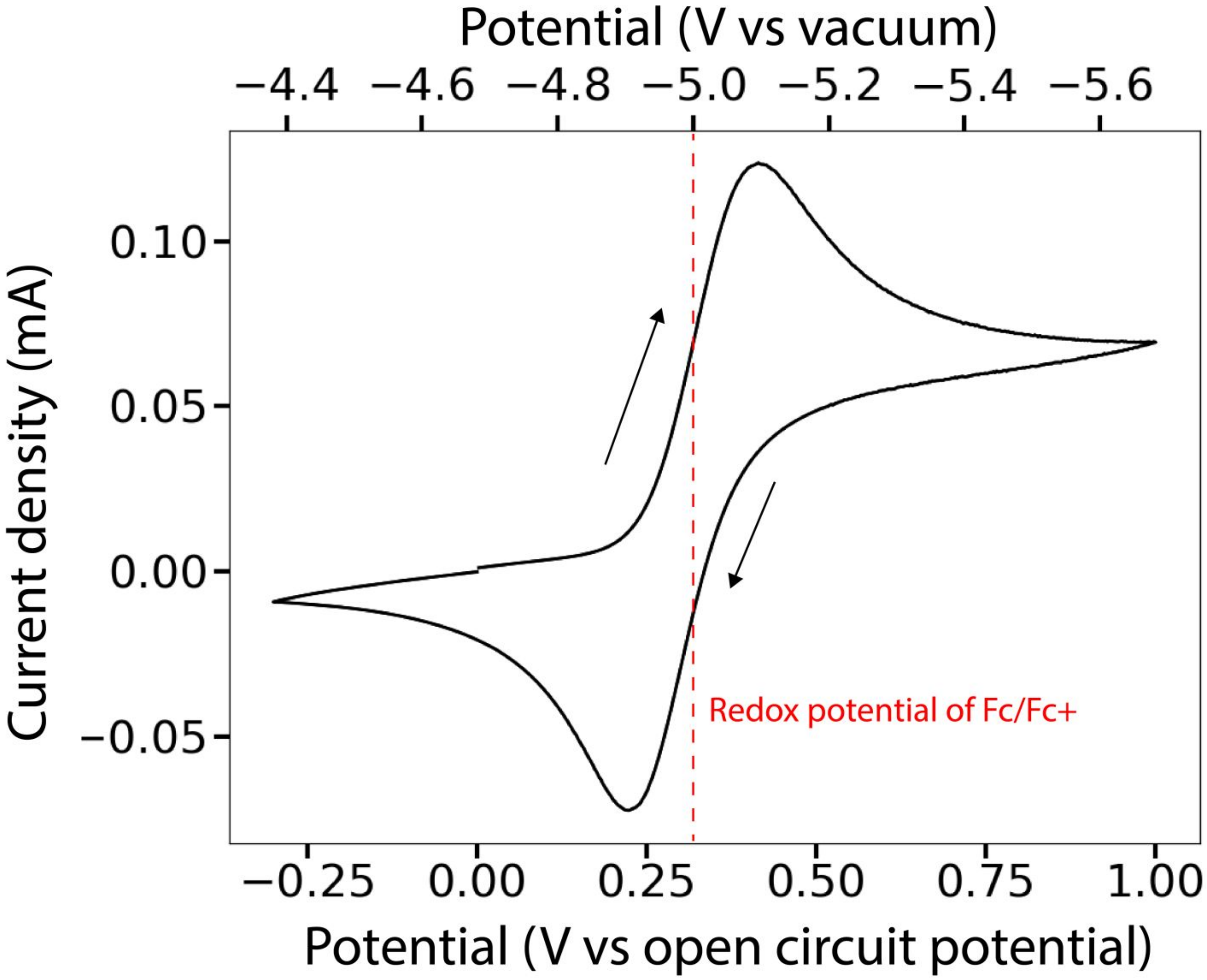

Figure S1. Experimental CV of the ferrocene/ferrocenium (Fc/Fc^+) pair in acetonitrile used for referencing in this work.[1] The redox potential of Fc/Fc^+ is at ~5.0 V *vs.* the vacuum level.[2]

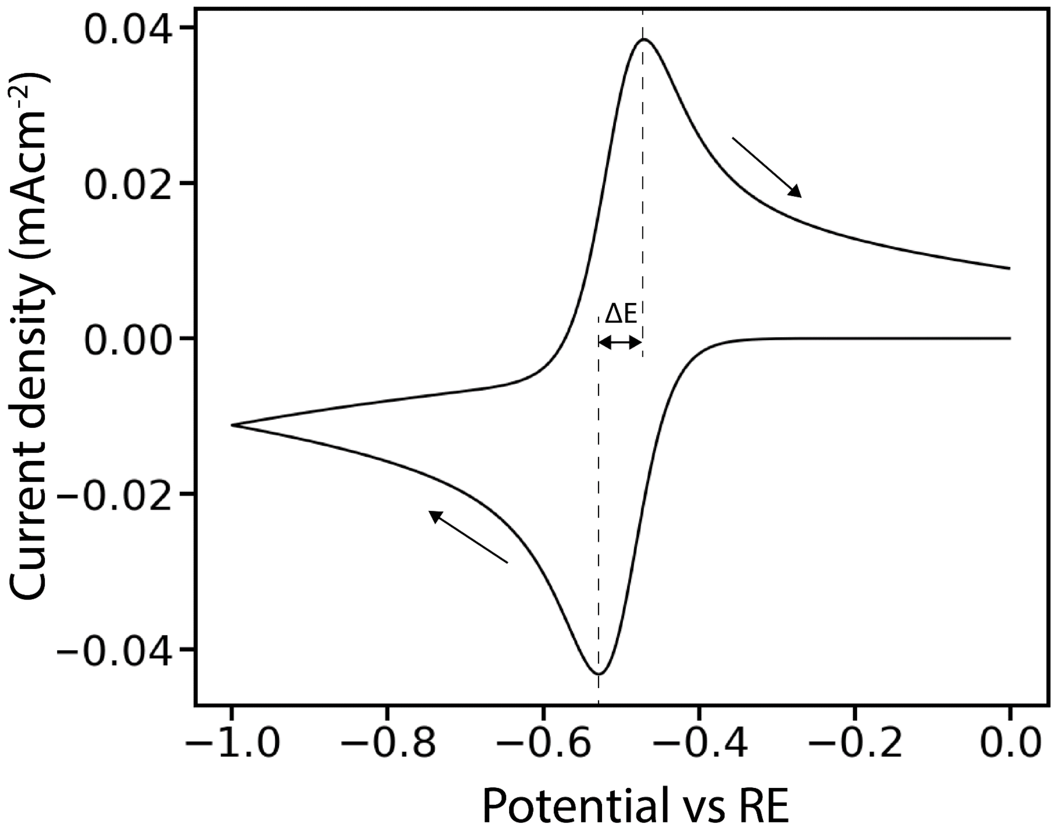

Figure S2. Simulated CV of a simple reductant/oxidant pair, E^0 = -0.5 V vs RE. As expected, ideal reversible behavior is observed with ΔE = 57 mV.

Table S2. List of parameters used in this work. Parameters used in CV simulations were chosen to reflect those of a ZnO QD film, then optimized to achieve a good fit with experimental results. Parameters for the steady-state simulations were chosen to achieve steady state quickly at a high spatial resolution.

Parameter	Value (CVs)	Value (steady-state)
QD film thickness	700 (nm)	70 (nm)
Number of lamella (film)	25	245
Lamella thickness (film)	27.94 (nm)	0.2837 (nm)
Number of lamella (film/solution interface)	65	
Lamella thickness (film/solution interface)	0.3 (nm)	0.1 (nm)
Distance between WE and CE	0.1 (mm)*	0.03 (mm)
Number of lamella (solution)	400	
Lamella thickness (solution)	~249 (nm)	~75 (nm)
Electron mobility	3e-10 ($m^2V^{-1}s^{-1}$)**	3e-11 ($m^2V^{-1}s^{-1}$)
Ion mobility (film)	3.5e-12 ($m^2V^{-1}s^{-1}$)	
Ion mobility (solution)	5e-10 ($m^2V^{-1}s^{-1}$)*	5e-11 ($m^2V^{-1}s^{-1}$)
Ion concentration (solution)	0.1 (M)	
Film porosity	50%	
Temperature	300 (K)	
Open circuit potential	-4.7 eV vs vacuum = 0.26 V vs SHE	
Relative permittivity electrolyte solution	37#	
Relative permittivity QD film	10	

* In reality, the ion mobility in the solution is expected to be higher, while the distance between the WE and CE is larger. For performance reasons, the mobility of ions was reduced. The distance between the WE and CE is lowered in accordance to avoid the formation of a large Ohmic drop over the solution. The mobility of the ions in solution is not limiting the injection of electrons, and thus increasing it further will not affect the results of the simulation. Performing the simulation with both 10 times increased ion mobility and distance between the WE and CE gave the same results, but took 10 times longer.

** It has been shown that electrons have a much higher mobility in ZnO QD films than the one used here.[1] Increasing the electron mobility higher than ~2 orders of magnitude above the ion mobility in the film does not affect the results of the simulation however, as cation mass transport in the film quickly becomes the limiting factor. For performance reasons, the electron mobility was therefore kept at an unphysically low level.

Based on acetonitrile. The QD films is assumed to be a mixture of a QD material and the electrolyte solution, so an arbitrary in-between value was picked for its relative permittivity. The relative permittivity of either the film of solution does not affect the results of the simulation as long as the spatial resolution is sufficient and can be put to any desired value.

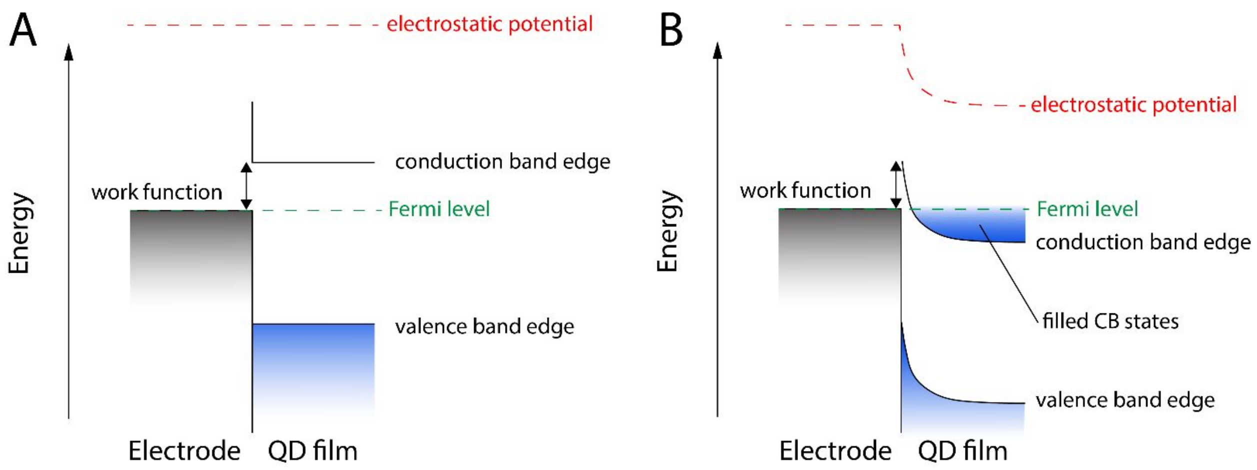

Figure S3. Energy level diagrams of the electrode and QD film at (A) open circuit potential and (B) negative applied potential > electron injection barrier. As the applied potential is increased, an electric double layer (EDL) develops at the QD film/electrode interface, causing the electrostatic potential to drop over the interface. This potential profile causes bend bending. When the applied potential is larger than the electron injection barrier (= conduction band edge – electrode work function), electrons can tunnel through the EDL into the conduction band states. The energy cost of entering a higher energy state (the semiconductor environment) is compensated by the gain in energy through the electrostatic potential.

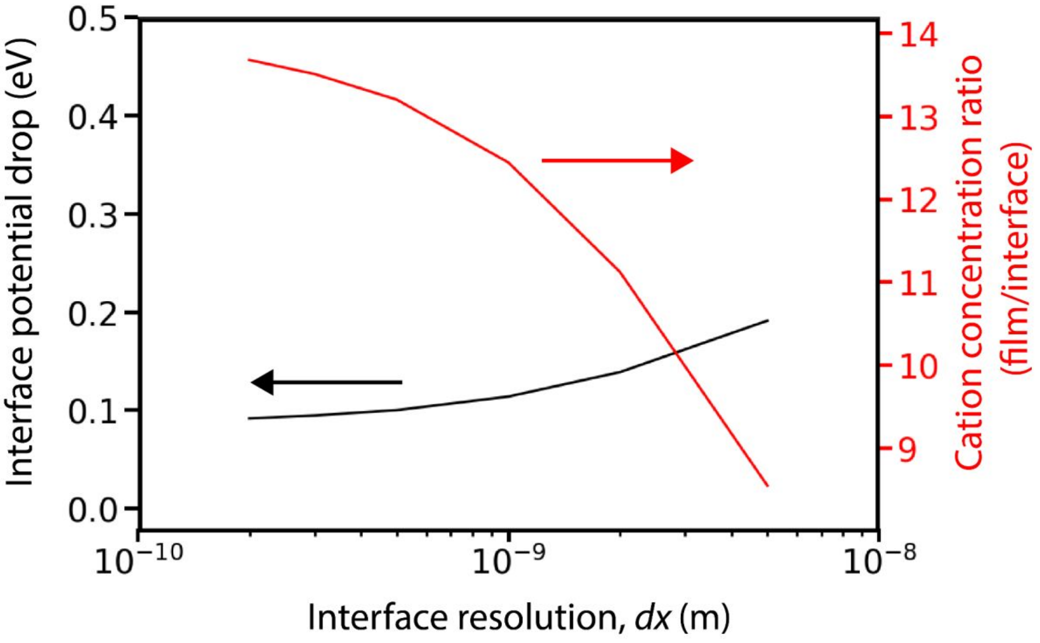

Figure S4. Steady-state interface potential drops and cation concentration ratios for different interface resolutions. The cation concentration ratio is calculated by dividing the concentration of cations in the film by the electrolyte concentration. When the resolution across the interface is insufficient, the simulator overestimates the interface potential drop and underestimates the cation concentration and thus doping density in the QD film. A resolution of 0.3 nm was deemed precise enough while still allowing for reasonable performance and was used in CV simulations.

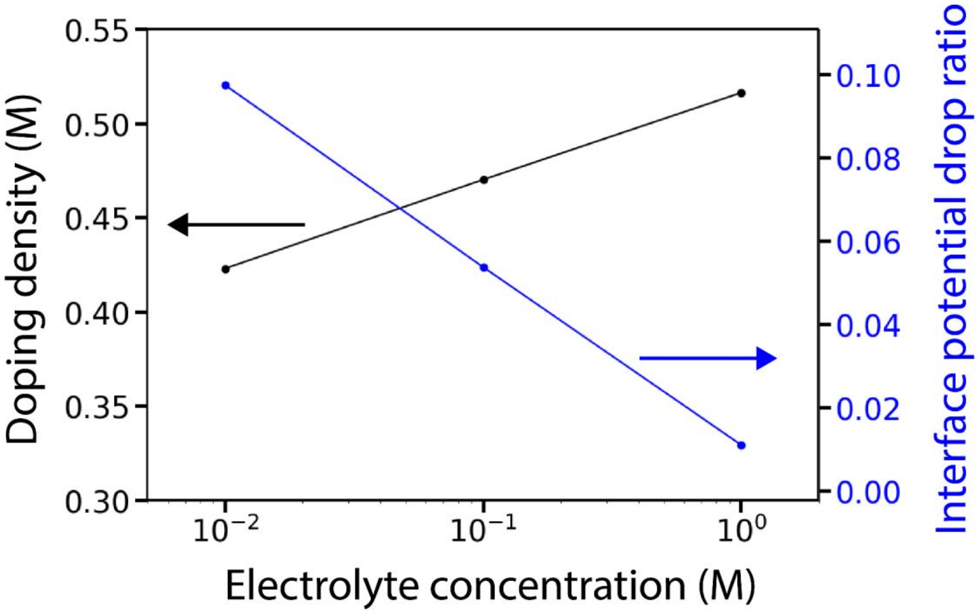

Figure S5. Steady-state interface potential drops and doping densities for different electrolyte concentrations. The interface potential drop is plotted as the ratio of the total potential drop (potential drop over the film/solution interface)/(voltage at WE vs RE). For lower electrolyte concentrations, the interface drop is larger and the steady state doping density of the QD film is lower.

Derivation S1: relation between the interface potential drop and the concentration of excess charge in the film.

Starting from the Boltzmann distribution:

$$c = c_0\, exp\, (\frac{-q\phi}{kT}) \qquad (1)$$

and $\frac{c_{film}}{c_0} = exp\, (\frac{q\Delta\phi_{F/S}}{kT})$ with $c_{film} = \frac{n}{p}$, assuming no anions are left in the film

Where c is the cation concentration, c_0 is the bulk electrolyte concentration (assuming an infinite supply of bulk cations), q is the elementary charge, ϕ is the electrostatic potential, k is the Boltzmann constant, T is the temperature, $\Delta\phi_{F/S}$ is the electrostatic potential drop over the film/solution interface, c_{film} is the concentration of cations in the electrolyte in the nanoporous film, n is the concentration of electrons in the film (for a QD film this is equal to the number of additional electrons per QD/the QD volume*the porosity of the film), and p is the porosity of the film. We can then define a minimum bulk concentration to keep $\Delta\phi_{F/S}$ below a certain threshold:

$$c_0 = \frac{n}{p * exp\left(\frac{q\Delta\phi_{F/S}}{kT}\right)} \qquad (2)$$

For example for a film (porosity = 50%) of QDs with a diameter of 3.5 nm (QD volume = 22 nm^3, QD “concentration” = 0.0742 M), if we wish to charge the film with 8 electrons per QD while keeping $\Delta\phi_{F/S} < 2$ kT (= 0.052 eV at RT), this would require a minimum electrolyte concentration of 0.16 M:

$$c_0 = \frac{0.594\ M}{0.5 * exp\ (2)} = 0.16\ M \tag{3}$$

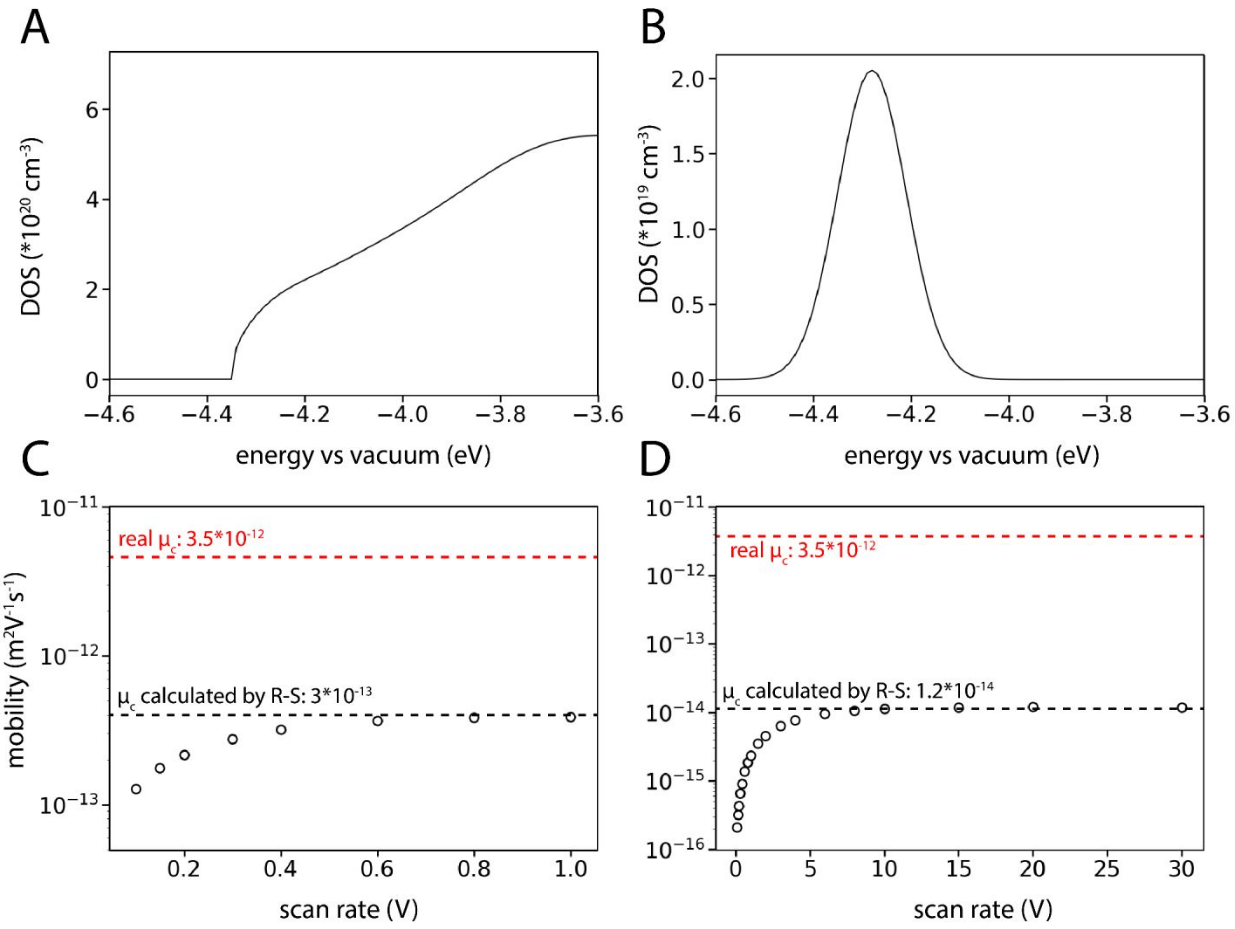

Figure S6. Cation mobilities (μ_c) in the QD film as calculated by the Randles-Ševčík equation compared to the mobility that was set at the start of the simulation, using two different density of states (DOS) functions. (A) The typical ZnO DOS function that was used in all other simulations.

(B) A DOS function that more closely resembles a simple electrochemical reaction (single Gaussian peak), such as may be observed when charging a battery electrode. Note the scaling, as (B) has a much lower total amount of states available. Because of this a higher scan rate is needed to enter the cation transport-limited regime. (C) and (D) show the respective scan rates series of simulations using (A) and (B) as DOS functions. As the scan rate increases, the calculated mobilities converge, showing that the current density becomes limited by the cation mass transport in the film at these scan rates. Depending on the specific DOS function, the Randles-Ševčík equation underestimates the mobility by a factor 10-300 for these systems.

Derivation S2: Solution of drift-diffusion equations for the charging of a nanoporous film

Table S3. Assumptions to solve the concentration profile during cation mobility-limited charging.

**(A1)**	Charge neutrality is preserved locally in the film.
**(A2)**	The current density in the film is constant over distance (follows from (1)).
**(A3)**	The electron mobility is much higher than the cation mobility ($\mu_n >> \mu_c$).

**(A4)**	$n(x) = c(x)$ and thus $dn/dx = dc/dx$, where x is the distance, n is the electron concentration and c is the excess hole concentration.
**(A5)**	Boundary condition 1: No cations flow into the WE ($J_c(x{=}WE) = 0$)
**(A6)**	Boundary condition 2: No electrons flow out of the film ($J_n(x{=}IF) = 0$)
**(A7)**	dn/dt is independent of x.

First we consider assumption (A7). Since the amount of electrons is increasing at the same rate throughout the whole film, the current density of electrons across the film must show a linear profile with distance. This is always observed in typical charging simulations and can also be understood intuitively as any non-linear profile of electron current would lead to the accumulation of electrons at certain positions in the film. That accumulation would lead to increased diffusion away from those positions, until a stable concentration profile is obtained, where the concentration of electrons increases at the same rate everywhere in the film and assumption 7 is satisfied. The same is true for cations, except their flow direction is reversed. In combination with assumption (A2) and boundary conditions (A5) and (A6) we find linear current density profiles for electrons (J_n) and cations (J_c) across the film, as depicted in Figure S7.

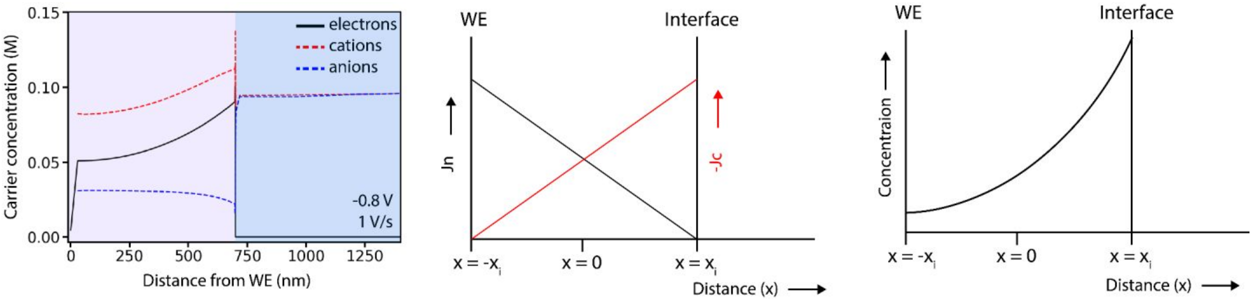

Figure S7. Concentration and current profiles in the QD film during cation-mobility limited charging.

Taking the midpoint between the WE and the IF as x = 0, so that $x_{IF} = x_i$ and $x_{WE} = -x_i$, we can then express the current density of electrons and holes as follows:

$$J_n(x) = \frac{J_{tot}}{2} + \frac{J_{tot} * x}{2x_i} \tag{4.A}$$

$$J_c(x) = \frac{J_{tot}}{2} - \frac{J_{tot} * x}{2x_i} \tag{4.B}$$

Where J_{tot} is the total current density over the film. We can then start from the drift-diffusion equations for electrons and excess cations:

$$J_n = nq\mu_n\frac{dV}{dx} - kT\mu_n\frac{dn}{dx} \tag{5.A}$$

$$J_c = cq\mu_c\frac{dV}{dx} + kT\mu_c\frac{dc}{dx} \tag{5.B}$$

Where n and c are the concentrations of electrons and cations, μ_i is the mobility of the respective carrier, V is the electric field, q is the elementary charge, k is Boltzmann's constant and T the temperature. We can substitute the current density profiles 1.A and 1.B to and rearrange to obtain:

$$nq\mu_n\frac{dV}{dx}+kT\mu_n\frac{dn}{dx}=\frac{J_{tot}}{2}-\frac{J_{tot}*x}{2x_i}$$

$$nq\mu_n\frac{dV}{dx}+=\frac{J_{tot}}{2}-\frac{J_{tot}*x}{2x_i}-kT\mu_n\frac{dn}{dx} \qquad (6.A)$$

$$cq\mu_c\frac{dV}{dx}-kT\mu_c\frac{dc}{dx}=\frac{J_{tot}}{2}+\frac{J_{tot}*x}{2x_i}$$

$$cq\mu_c\frac{dV}{dx}=\frac{J_{tot}}{2}+\frac{J_{tot}*x}{2x_i}+kT\mu_c\frac{dc}{dx} \qquad (6.B)$$

When we divide 3.A and 3.B, then apply assumption (4) we find:

$$\frac{\frac{J_{tot}}{2}-\frac{J_{tot}*x}{2x_i}-kT\mu_n\frac{dn}{dx}}{\frac{J_{tot}}{2}+\frac{J_{tot}*x}{2x_i}+kT\mu_c\frac{dc}{dx}}=\frac{nq\mu_n\frac{dV}{dx}}{cq\mu_c\frac{dV}{dx}}$$

$$\frac{\frac{J_{tot}}{2}-\frac{J_{tot}*x}{2x_i}-kT\mu_n\frac{dc}{dx}}{\frac{J_{tot}}{2}+\frac{J_{tot}*x}{2x_i}+kT\mu_c\frac{dc}{dx}}=\frac{cq\mu_n\frac{dV}{dx}}{cq\mu_c\frac{dV}{dx}}=\frac{\mu_n}{\mu_c} \qquad (7)$$

$$\left(\frac{J_{tot}}{2}-\frac{J_{tot}*x}{2x_i}-kT\mu_n\frac{dc}{dx}\right)\mu_c=(\frac{J_{tot}}{2}+\frac{J_{tot}*x}{2x_i}+kT\mu_c\frac{dc}{dx})\mu_n$$

$$\mu_c J_{tot}\left(\frac{1}{2} - \frac{x}{2x_i}\right) + kT\mu_c\mu_n\frac{dc}{dx} = \mu_n J_{tot}(\frac{1}{2} - \frac{x}{2x_i}) - kT\mu_c\mu_n\frac{dc}{dx}$$

$$\mu_c J_{tot}\left(\frac{1}{2} - \frac{x}{2x_i}\right) - \mu_n J_{tot}(\frac{1}{2} - \frac{x}{2x_i}) = 2kT\mu_c\mu_n\frac{dc}{dx}$$

$$\frac{J_{tot}}{2}\left((\mu_c - \mu_n) - \frac{x}{x_i}(\mu_c - \mu_n)\right) = 2kT\mu_c\mu_n\frac{dc}{dx}$$

$$\frac{dc}{dx} = \frac{\frac{J_{tot}}{2}\left((\mu_c - \mu_n) - \frac{x}{x_i}(\mu_c - \mu_n)\right)}{2kT\mu_c\mu_n} = x\frac{J_{tot}(\mu_c - \mu_n)}{4x_i kT\mu_c\mu_n} + \frac{J_{tot}(\mu_c - \mu_n)}{4kT\mu_c\mu_n} \qquad (8)$$

After integration of *c* to *dx* we find a quadratic concentration profile for cations and thus also for electrons because of assumption A4 (Figure S7C):

$$c(x) = -x^2\frac{J_{tot}(\mu_c - \mu_n)}{8x_i kT\mu_c\mu_n} + x\frac{J_{tot}(\mu_c - \mu_n)}{4kT\mu_c\mu_n} + c_0 \qquad (9)$$

Furthermore by calculating $\Delta c = c(xi) - c(-xi)$ we find:

$$\Delta c = -x_i^2\frac{J_{tot}(\mu_c - \mu_n)}{8x_i kT\mu_c\mu_n} + 2x_i\frac{J_{tot}(\mu_c - \mu_n)}{4kT\mu_c\mu_n} + x_i^2\frac{J_{tot}(\mu_c - \mu_n)}{8x_i kT\mu_c\mu_n} = 2x_i\frac{J_{tot}(\mu_c - \mu_n)}{4kT\mu_c\mu_n} \qquad (10)$$

$$J_{tot} = \frac{4kT \quad \mu_c \mu_n}{2x_i \Delta c(\mu_c - \mu_n)}$$

Considering *2xi* = film thickness *L*, we come to the following expression for the current density at any given point in the QD film during diffusion-limited charging:

$$J_{tot} = \frac{4kT \quad \mu_c \mu_n}{L\Delta c(\mu_c - \mu_n)} \tag{11}$$

As expected, the current density is inversely proportional to the concentration gradient and the thickness of the film. If one of the charge carriers is limiting (as in this case the cations are), the expression reduces to:

$$J_{tot} = -\frac{4kT\mu_c}{L\Delta c} \tag{12}$$

And a simple linear relationship between current density and charge carrier mobility is observed. Unfortunately, since Δc (or Δn) is unknown during an experiment and depends on many factors, we cannot directly use this expression to determine the limiting mobility during a CV scan. It should be noted that while the charge transport in this case is limited by the mobility of the cations and charging of the film is in a diffusion-limited regime, drift current cannot be neglected. This

follows logically from assumptions (A2), (A3) and (A4): since the concentration profile of both carriers is the same, their diffusion fluxes point in the same direction (both electrons and cation diffuse towards the WE). However, electrons necessarily must move in the opposite direction as cations during film charging. To achieve this, an electric field is needed, which is always present in the film during charging. Thus both drift and diffusion are contributing to the overall current in the film.

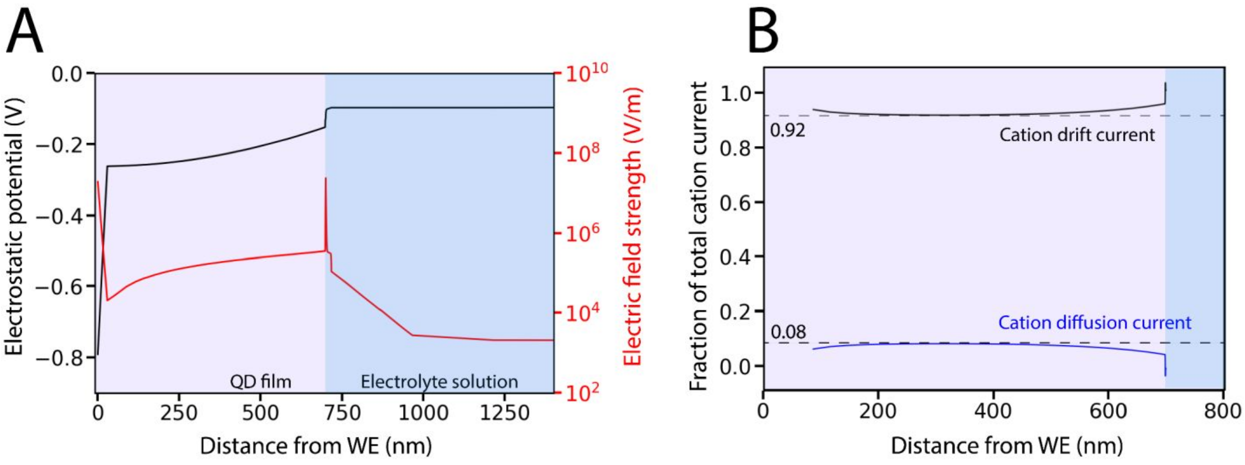

Figure S8. The potential and electric field in the QD film during cation transport-limited charging. A strong electric field is present in the QD film even during cation transport-limited charging, which leads to drift currents in the film.

References for the supporting information

1. Gudjonsdottir, S.; Van Der Stam, W.; Kirkwood, N.; Evers, W. H.; Houtepen, A. J., The role of dopant ions on charge injection and transport in electrochemically doped quantum dot films. *Journal of the American Chemical Society* **2018,** *140* (21), 6582-6590.
2. Bard, A. J.; Faulkner, L. R., *Fundamentals and applications*. 2001; Vol. 2, p 580-632.
